# A Comparative Study of Oral Health-Related Quality of Life among Cleft Lip and Palate Patients and Their Families during Orthodontic Treatment

**DOI:** 10.3390/ijerph182312826

**Published:** 2021-12-05

**Authors:** Inês Francisco, Francisco Caramelo, Maria Helena Fernandes, Francisco Vale

**Affiliations:** 1 Institute of Orthodontics, Faculty of Medicine, University of Coimbra, 3000-075 Coimbra, Portugal; 2 Institute of Clinical and Biomedical Research of Coimbra (iCBR), Faculty of Medicine, University of Coimbra, 3000-075 Coimbra, Portugal; fcaramelo@fmed.uc.pt; 3 Faculty of Dental Medicine, University of Porto, 4200-393 Porto, Portugal; mhfernandes@fmd.up.pt; 4 LAQV/REQUIMTE, University of Porto, 4160-007 Porto, Portugal

**Keywords:** quality of life, orthodontics, cleft palate, family, caregiver

## Abstract

(1) Background: It has been recognized that CLP condition may affect oral health-related quality of life (OHRQoL) due to dental anomalies and abnormal craniofacial development. Aim: To assess whether orthodontic treatment affected the levels of OHRQoL in CLP patients and their families. (2) Methods: 226 individuals (111 with cleft and 115 control) and their parents were invited to complete the Oral Health Impact Profile-14 (OHIP-14) and Family Impact Scale (FIS), respectively. The Mann–Whitney test was used for quantitative variables and the Fisher’s exact test for categorical variables. The Spearman Rank Correlation Coefficient was used to correlate the results of the OHIP and FIS questionnaires. (3) Results: No significant difference was found between groups in OHIP-14 but FIS score revealed a significant difference between the two groups evaluated (*p* < 0.001). Only the social limitation in OHIP score revealed a significant difference (*p* = 0.001). Regarding FIS score, the most affected dimensions were family activities (*p* < 0.001), parental emotions (*p* = 0.001), and family conflict (*p* = 0.011). (4) Conclusion: Undergoing orthodontic treatment had a similar impact on the overall quality of life in CLP patients and non-cleft patients. Parents of cleft children had a poorer OHRQoL compared to what was perceived by their children and parents of non-cleft children.

## 1. Introduction

Cleft lip and palate is a congenital condition that results from failure of embryonic processes in the fifth and tenth weeks of intrauterine life [1]. The etiology includes several genetic (e.g., IRF6, VAX1 and PAX7) and environmental risk factors (e.g., smoking consumption during pregnancy and gestational diabetes). Moreover, several studies aimed to estimate the main effects of genes-environment interaction, but the results of these interactions are inconclusive due to methodological flaws, namely low statistical power analyses, differences in genotyped samples and differential assessment of environmental exposures [2,3,4]. Treatment of patients with cleft lip and palate (CLP) is considered one of the most challenging, since CLP leads to several difficulties such as feeding, hearing, speaking, breathing and dentofacial development [1]. The presence of dental anomalies and abnormal craniofacial development can lead to the development of malocclusion such as crossbite (anterior or posterior), open bite, skeletal Class III and crowding [5]. Dental anomalies are significantly more frequent in CLP patients than general population, leading to a long-term impact on patient’s facial anatomy and self-esteem [6].

Over the past few years, it has been recognized that CLP condition may affect oral health-related quality of life (OHRQoL) [7]. In an earlier study, Montes et al. showed that unilateral CLP patients reported more orofacial dysfunctions and negative impacts on social well-being than controls [8]. Age, sex and cleft phenotype influence the OHRQoL score. Typically, older ages and female had a higher impact on OHRQoL in CLP patients, but these results are still controversial in the literature [9]. Moreover, CLP patients can experience lower OHRQoL in physical pain and psychological discomfort domains despite the comprehensive treatment received [10]. The rehabilitation of CLP patients requires an interdisciplinary team, in which the orthodontist plays a fundamental role from birth to adulthood.

As for the orthodontic treatment, it has been shown in the literature that it can influence OHRQoL: Malocclusion in aesthetic zone was associated with worst OHRQoL score [11]; Patients with worst OHRQoL are more likely to seek further treatment than patients with high OHRQoL [12]; Children with low psychological well-being can benefit from orthodontic treatment [13]; Adverse side effects of orthodontic treatment may decrease OHRQoL, such as pain and anxiety [14]; Patients who had completed orthodontic treatment had a better OHRQoL than those under treatment or who never had treatment [15]; During orthodontic treatment, OHRQoL is worse in oral symptoms and functional limitations domains but better in emotional well-being [16].

A recent review of outcomes related to orthodontic treatment in CLP patients found that quality of life and health resource use are the outcomes with the least representativeness in the literature [9]. To date, most studies in this field found that surgical patients had a significantly greater reduction in OHRQoL score than patients with severe malocclusions or cleft patients [17]. Barros et al. compared Class III malocclusion with unilateral CLP and suggested that OHRQoL appears to be more affected by the etiology of Class III than by surgical or orthodontic treatment [18]. Despite this, the impact of orthodontic treatment on CLP patients without considering surgical approaches remains ambiguous.

Additionally, the continuous demands imposed on the caregiver during CLP patient’s rehabilitation influence caregivers’ well-being. The impact on OHRQoL appears to be similar between CLP patients and their caregivers [19]. Beluci et al. reported that caregivers with greater burdens have a worse perception of the quality of life. They also described that the perception of rehabilitation and the coping capacity of CLP children are influenced by the expectations, attitudes and support of their parents [20]. Difference on OHRQoL scores by children and their parents were reported with parents underestimate the “Oral symptoms” but overestimate “Functional Well-Being” of having a cleft [21]. Psychologic events, such as stigma and discrimination among peers due to CLP condition, may also affect the quality of life of patients and their families [22]. Moreover, Raghavan et al. found that parents rated the malocclusion more critically than CLP patients [23]. The effect of orthodontic treatment in patients without craniofacial disharmony is accepted to be highly beneficial on the families [24]. Therefore, the rehabilitation of CLP patients must also focus on family’s needs approach. As far we are aware, no study had evaluated OHRQoL of parents regarding CLP children undergoing orthodontic treatment.

Knowledge of OHRQoL improves treatment quality, since it helps to identify the functional, emotional and social impacts, contributing to the planning of health care and clinical interventions [7]. This is even more important in CLP patients since they have a lengthy orthodontic treatment. Despite the increase in the number of studies involving QoL and orthodontic treatment, there is little evidence in the evaluation of the impact of orthodontic treatment on CLP patients and their families. The aim of this study was to assess if the condition of having a cleft affected the levels of OHRQoL in patients undergoing orthodontic treatment and their families. The following hypothesis are proposed:

1. There are differences in Oral Health Impact Profile-14 between patients with CLP and healthy controls (non-cleft group) undergoing orthodontic treatment.
2. There are differences in Family Impact Scale between parents of children with CLP and parents of healthy children (non-cleft group) who are undergoing orthodontic treatment.
3. There are differences in the perception of the quality of life between patients and their parents.
4. Age and sex influence the perception of the OHRQoL in CLP and healthy controls groups undergoing orthodontic treatment.

## 2. Materials and Methods

### 2.1. Study Design

This cross-sectional study was approved by the Ethics Committee of Faculty of Medicine of University of Coimbra (number CE-071/2020) in accordance with the Declaration of Helsinki. All participants, or parents when applicable, were instructed on the research and provided a written informed consent. This study was reported according to the CONSORT (Consolidated Standards of Reporting Trials) guideline.

### 2.2. Data Collection Procedure

The sample size was based on previous studies that investigate the effects of orthodontic treatment on quality of life [15]. Prior data indicated an increase in OHRQoL score from 23.0 to 29.3 in patients undergoing orthodontic treatment. We assumed that this difference would be the minimum value to be considered to be clinically relevant and, thus that the two groups under study should differ at least of 6.3. To compute the effect size, a standard deviation of 11 was assumed. The initial calculated sample size was estimated using G*Power 3.1.9.2 considering a bilateral independent *t*-test, with an 80% power in the detection of differences and 5% level of significance. The number of subjects obtained was 48 but taking a 20% for loss to follow-up and dropouts into consideration, a final required sample size was 58 for each group. In the present work, the sample included a higher number of cleft and control individuals, allowing high confidence in the results.

During the data collection (July 2019 to February 2021), all patients in active orthodontic treatment with fixed multibracket for at least 6 months and less than 10 months from the aforementioned Institution were invited to participate in this study. The sample was selected according to the following inclusion criteria:

- The Cleft group is composed by individuals from both sexes with cleft lip and palate undergoing orthodontic treatment in the Faculty of Medicine of the University of Coimbra.
- The Control group is composed by individuals who attended the Faculty of Medicine of the University of Coimbra for orthodontics care without cleft lip and palate condition. The same number of patients was selected as control group, using randomized sampling resorting to a simple random allocation process with adaptive probabilities to guarantee a match, regarding age and sex, between the groups. This process aimed to ensure a reliable and unbiased comparison between the control and the CLP group.

The exclusion criteria were patients with cognitive disorders, craniofacial syndromes, multiple dental loss, untreated dental caries, periodontal disease, severe facial trauma, chronic pain and patients who previously underwent orthodontic treatment.

### 2.3. Questionnaires

OHRQoL was assessed by 2 standardized instruments: Oral Health Impact Profile-14 (OHIP-14) and Family Impact Scale (FIS) (Table A1 and Table A2, respectively). Patients and parents were invited to complete the questionnaire after the routine orthodontic consultation in the waiting room and then return it in a sealed envelope to an individual not involved in the study.

The children’s ORHQoL was assessed using the OHIP-14. OHIP-14 consists of 14 questions arranged over 7 domains: functional limitations, physical pain, psychosocial impact, physical limitation, psychological limitation, social limitation and disability. Participants were asked to rate the frequency of an event on a 5-point Likert scale: never = 0; once/twice = 1; sometimes = 2; often = 3; every day/almost day = 4. The sum of the responses allows the evaluation of total OHIP scores and individual OHRQoL domain. A high OHIP-14 score corresponds a high negative impact on the OHRQoL. Participants were instructed to answer the questions without any support from their parents and if they have any doubt, clinical staff helps them. The age, sex and presence of cleft were also registered.

The FIS was used to evaluate the impact of a child’s oral condition on family life. The FIS is composed of 14 questions into 4 domains: parental activities, parental emotions, family conflict and family finances. The questions have five Likert response options: never = 0; once/twice = 1; sometimes = 2; often = 3; every day/almost day = 4. Summating all questions, ranging from 0 to 56, derives an overall FIS score. A higher FIS score indicates a greater impact of a child’s oral condition on family.

### 2.4. Statistical Analysis

Data were analysed using the Statistical Package for the Social Sciences, version 24.0 for Windows (SPSS Inc., Chicago, IL, USA). The significance level adopted for all analyses was 0.05. Descriptive statistics for the quantitative variables were obtained using mean, standard deviation values and minimum and maximum. Categorical variables were expressed as absolute and relative frequency.

The comparison between the groups was performed using the Mann–Whitney test for quantitative variables, when was verified a violation of the normality assumption through the Shapiro–Wilk test. The Fisher’s exact test was used to verify statistical differences between groups for categorical variables.

The Spearman Rank Correlation Coefficient was used to correlate the results of the OHIP and FIS questionnaires. Linear regression was performed to correlate sex and age with the OHIP questionnaire.

## 3. Results

A total of 226 children or young adults (111 with cleft lip and palate and 115 healthy individuals) and their parents completed the OHIP-14 and FIS, respectively. Patients were in the age range of 8–27 years and homogenous individuals matched population was obtained regarding sex and age (Table 1). No statistically significant difference was found between both groups and sex and age variables (Table 2). The CLP group comprises 86 (77.5%) individuals with unilateral cleft and the remaining 25 (22.5%) had bilateral cleft.

**Figure 1:**
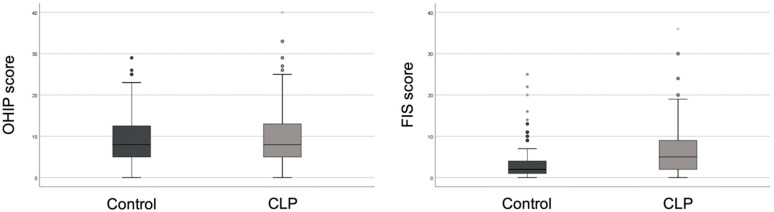
Distribution of values in OHIP and FIS scores.

Regarding the global questions, the mean score for quality of life according to the OHIP was 9.4 ± 6.2 and 10.2 ± 7.2 for the control and CLP group, respectively. The parent-reported overall FIS score was 4.0 ± 5.4 for the control group and 6.7 ± 6.3 for the cleft group. No significant difference was found between groups in OHIP-14 score. Conversely, FIS score revealed a significant difference between the two groups evaluated (*p* < 0.001) (Table 3). The distribution of values in OHIP and FIS scores is shown in Figure 1. Regarding the type of cleft (unilateral vs bilateral) the global scores do not have statistically significant differences (OHIP-14: *p* = 0.382; FIS; *p* = 0.873).

**Figure 2:**
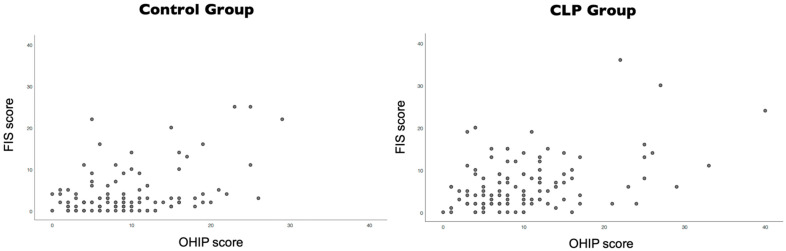
Correlation between patients’ and parents’ quality-of-life assessment.

There was a weak positive correlation between a patient’s quality of life (OHIP) and a parent’s quality of life (FIS) assessment. Additionally, it was also verified that control group (r_Spearmann_ = 0.267, *p* = 0.004) had a lower correlation than the CLP group (r_Spearmann_ = 0.328, *p* < 0.001). Figure 2 shows dispersion graphs of both groups.

The analysis of the different OHIP domains revealed that only the social limitation domain shows a significant difference between the two groups (*p* = 0.001), with CLP patients presenting the highest score. Regarding FIS score, the most affected dimensions were family activities (*p* < 0.001), parental emotions (*p* = 0.001) and family conflict (*p* = 0.011). In all these three domains, parents with a cleft child had the highest scores than the control group. Table 4 shows the scores in the different OHIP and FIS domains by the two groups.

The analysis of the correlation between age or sex on quality-of-life impacts showed that both variables do not affect the perception of OHIP score (sex: *p* = 0.170; age: *p* = 0.406).

## 4. Discussion

This study evaluated the quality of life in 226 children with and without CLP and their parents with two validated indexes (OHIP-14 and FIS) during orthodontic treatment. It was found that children had a similar OHRQoL but the family impact was highest in parents of cleft children.

Malocclusion has been reported to play a negative role on quality of life [11]. Since CLP patients usually have malocclusion due to dental anomalies and abnormal craniofacial development, it would be expected that CLP patients presented worse scores of OHRQoL [5]. Despite that, this study verified no significant difference in OHIP-14 score, which is in line with previous studies [25,26]. Aravena et al. reported similar OHRQoL on CLP and control children despite the cleft group had a lower quality-of-life score concerning speech items [26]. Additionally, the current study included cleft patients undergoing orthodontic treatment, demonstrating that OHRQoL score remains similar between groups. In the literature, a positive effect after orthodontic treatment in healthy and CLP patients was reported [24,27]. Chen et al. showed that completed orthodontic treatment had a positive effect on the quality of life, especially when orthodontic surgical treatment was performed [27].

The social limitation was the only OHIP domain with a significant difference between the two groups (*p* = 0.001), with CLP patients presenting the highest score. This result is in accordance with the study of Sundell et al. which verified that the 10-year-old children with CLP perceived lower OHRQoL than the non-cleft controls [28]. Khoun et al. found that the cleft group had a greater impact on emotional and social well-being compared to the others study groups (severe caries; malocclusion; and dentine caries) [29]. Several explanations for this finding were given, namely CLP children feel less acceptance by peers due to the facial appearance and hypernasal speech; CLP children have less ability to maintain relationships; and CLP children may have fewer experiences of positive group feeling [28].

The family’s perspective should also be assessed because chronic illness, such as cleft lip and palate, inevitably impacts the day-to-day functioning since health care interventions will lead to family needs and concerns [30]. Family activities, parental emotions and family conflict were identified as significant factors negatively affecting parents OHRQoL in this total sample. Previous studies pointed out some factors that may explain the impact on quality of life of caregivers, namely repeated consultations, financial implications, parental time off work, less time for other family members, stigmatization and peer victimization of their child and the impact of orthodontic appliances in speech, mastication and social interaction of the child [30,31]. On the other hand, the family finances domain did not show a significant difference between the two groups (*p* = 0.051). Cuyper et al. found that parents of non-syndromic children had more family conflicts, suggesting that having a cleft child entails financial implications and decreased participation in social activities [31]. These distinct findings may be explained by this cross-sectional study having a sample recruited from a university hospital environment where the treatments provided are supported by the National Health Service. Therefore, this sample may be limited in representing the population in this domain, as the recruitment to the university hospital depends on the eligibility for funding.

Concerning a patient’s quality of life (OHIP) and a parent’s quality of life (FIS) assessment, it has been verified a weak positive correlation. Parents of cleft children had a poorer OHRQoL compared to what was perceived by their children. Several reasons for this finding have been suggested, namely the overestimation of the cleft implications in children social integration at early ages, guilty feelings, and better understanding of the impact of cleft on children’s development, which can lead to a higher burden of concerns [25,32]. Additionally, Imani et al. suggested that parents of CLP children under orthodontic treatment are more vulnerable due to their previous adverse experiences throughout the treatment of their children [33].

Finally, no significant difference was found regarding the correlation between age (age: *p* = 0.406) or sex (sex: *p* = 0.170) on quality-of-life impacts. This not corroborated earlier studies that showed that the higher the age the higher the impact on quality of life and that females generally have a greater impact on OHRQoL [19]. The results obtained in this study can be explained by the homogenous age and sex group distribution of the sample. Recently, Agnew et al. demonstrated no difference in FIS score by age, sex or whether a child had started orthodontic treatment [30].

This study presents some limitations that need to be discussed. First, the scores used were not developed to evaluate craniofacial deformities. CLP patients have several problems that are not directly assessed by the scores used, such as facial and speech features [12]. Second, the answers to the questionnaire can be influenced by other aspects of life that are not necessarily related to craniofacial deformity. However, the scores (OHIP-14 and FIS) used in this study are widely used in dentistry to measure the quality of life, permitting the comparison of the findings of this study with other studies. Moreover, these scores had validation in the native language of this sample, and it has previously been shown that OHIP-14 is a good method to measure OHRQoL due to its simplicity and good discriminative properties in patients with simple and complex treatment (e.g., dentofacial deformities) [17]. Third, no distinction between patients regarding the severity of the cleft or malocclusion was performed, which could potentially introduce some bias. Therefore, the results obtained should be considered with caution as they might not be completely representative of the cleft lip and palate population. Nevertheless, Sundell et al. reported no differences between cleft type and overall mean score [28]. Fourth, the age range of 27-8 years may influence the results obtained since this study used self-reported questionnaires which can lead to several problems, namely children’s abilities to read or speak and their capacity to understand abstract terms used in the questionnaire [34].

Despite these limitations, this study presents some strengths such as adequate sample power with a homogenous distribution, allowing a reduction of the bias of the analysed data. This study presents novel insights into the impact of orthodontic treatment in quality of life of patients and their families, which may contribute to the communication among physicians, patients and parents/caregivers. Further studies should establish greater insight into specific factors influencing quality of life such as the severity of cleft or malocclusion. Moreover, the effects of various phases of orthodontic treatment should be studied.

## 5. Conclusions

Undergoing orthodontic treatment had a similar impact on the overall quality of life in CLP patients and non-cleft patients. Parents of cleft children had a poorer OHRQoL compared to what was perceived by their children and parents of non-cleft children. No significant difference was found regarding the age and sex of the child.

## Figures and Tables

**Table 1:** Age and sex by each group.

	CLP Group (*n* = 111)		Control Group (*n* = 115)		
Year of Birth	Female	Male	Female	Male	Total
1994	1	0	1	0	2
1995	1	1	1	1	4
1996	0	1	0	1	2
1997	3	1	3	1	8
1998	1	3	1	3	8
1999	0	1	0	1	2
2000	3	2	3	2	10
2001	3	2	3	2	10
2002	4	4	4	4	16
2003	5	4	5	4	18
2004	5	8	5	6	24
2005	0	6	0	8	14
2006	5	8	5	7	25
2007	6	3	6	3	18
2008	1	3	1	3	8
2009	3	8	3	8	22
2010	1	7	1	12	21
2011	2	1	2	1	6
2012	1	0	1	0	2
2013	0	3	0	3	6
Total	45	66	45	70	226

**Table 2:** Demographic statistics for the sample studied.

Demographic Variables	Control (115)	CLP (111)	*p*
sex (M/F)	70/45 (60.9%/39.1%)	66/45 (59.5%/40.5%)	0.892 ^§^
$\mathrm{age}\;\bar{x}\pm \mathrm{sd}$ (min/max)	15.0 ± 4.3 (7/36)	15.3 ± 4.3 (7/26)	0.637 ^£^

^§^ Fisher’s exact test; ^£^ Mann–Whitney; sd standard deviation.

**Table 3:** Demographic statistics for the sample studied.

OHIP and FIS Scores	Control (115)	CLP (111)	*p*
OHIP $\bar{x}\pm \mathrm{sd}$ (min/max)	9.4 ± 6.2 (0/29)	10.2 ± 7.2 (0/40)	0.572 ^£^
FIS $\bar{x}\pm \mathrm{sd}$ (min/max)	4.0 ± 5.4 (0/25)	6.7 ± 6.3 (0/36)	<0.001 ^£^

^£^ Mann–Whitney; sd standard deviation.

**Table 4:** OHIP and FIS answers distribution regarding the domains.

		Control (115)	CLP (111)	*p* ^£^
**OHIP**	Functional limitation	1.3 ± 1.0 (0/4)	1.6 ± 1.4 (0/6)	0.192
	Physical pain	3.2 ± 1.7 (0/8)	3.0 ± 1.6 (0/7)	0.507
	Psychosocial impact	1.3 ± 1.5 (0/6)	1.5 ± 1.9 (0/8)	0.904
	Physical limitation	1.5 ± 1.8 (0/8)	1.3 ± 1.6 (0/7)	0.253
	Psychological limitation	1.0 ± 1.4 (0/7)	1.1 ± 1.6 (0/7)	0.846
	Social limitation	0.8 ± 1.1 (0/4)	1.4 ± 1.5 (0/6)	0.001
	Disability	0.3 ± 0.7 (0/4)	0.3 ± 0.9 (0/4)	0.531
**FIS**	Family activities	2.0 ± 2.6 (0/12)	3.7 ± 3.2 (0/18)	<0.001
	Parental emotions	0.7 ± 1.6 (0/8)	1.3 ± 2.1 (0/12)	0.001
	Family conflict	0.7 ± 1.6 (0/7)	1.2 ± 2.0 (0/9)	0.011
	Family finances	0.6 ± 0.8 (0/4)	0.4 ± 0.9 (0/3)	0.051

^£^ Mann–Whitney.

## Data Availability

The data presented in this study are available on request from the corresponding author.

## Appendix A

**Table A1:** OHIP-14 Questionnaire. * O = never, l = hardly ever. 2 = occasionally 3 = fairly often, 4 = very often.

Dimension	Question	Response *
Functional limitation	Have you had trouble pronouncing any words because of problems with your teeth, mouth or dentures?	
	Have you felt that your sense of taste has worsened because of problems with your teeth, mouth or dentures?	
Physical pain	Have you had painful aching in your mouth?	
	Have you found it uncomfortable to eat any foods because of problems with your teeth, mouth or dentures?	
Psychological discomfort	Have you been self-conscious because of your teeth, mouth or dentures?	
	Have you felt tense because of problems with your teeth, mouth or dentures?	
Physical disability	Has your diet been unsatisfactory because of problems with your teeth, mouth or dentures?	
	Have you had to interrupt meals because of problems with your teeth, mouth or dentures?	
Psychological disability	Have you found it difficult to relax because of problems with your teeth, mouth or dentures?	
	Have you been a bit embarrassed because of problems with your teeth, mouth or dentures?	
Social disability	Have you been a bit irritable with other people because of problems with your teeth, mouth or dentures?	
	Have you had difficulty doing your usual jobs because of problems with your teeth, mouth or dentures?	
Handicap	Have you felt that life in general was less satisfying because of problems with your teeth, mouth or dentures?	
	Have you been totally unable to function because of problems with your teeth, mouth or dentures?	

**Table A2:** FIS Questionnaire. * O = never, l = hardly ever. 2 = occasionally 3 = fairly often, 4 = very often.

Dimension	Question	Response *
Parental/family activity	Have you or has the other parent taken time off work?	
	Has your child required more attention from you or the other parent?	
	Have you or has the other parent had less time for yourself or other family members?	
	Has your sleep or that of the other parent been disrupted?	
	Have family activities been interrupted?	
Parental emotions	Have you or has the other parent been upset?	
	Have you or has the other parent felt guilty?	
	Have you or has the other parent worried that your child will have fewer life opportunities?	
	Have you felt uncomfortable in public places?	
Family conflicts	Has your child argued with you or the other parent?	
	Has your child been jealous of you or other family members?	
	Has your child´s condition caused disagreement or conflict in the family?	
	Has your child blamed you or the other parent?	
Financial Burden	Has your child´s condition caused financial difficulties for your family?	
